# Towards Accurate Estimation of the Proportion of True Null Hypotheses in Multiple Testing

**DOI:** 10.1371/journal.pone.0018874

**Published:** 2011-04-22

**Authors:** Shu-Dong Zhang

**Affiliations:** Centre for Cancer Research and Cell Biology (CCRCB), Queen's University Belfast, Belfast, United Kingdom; University of Georgia, United States of America

## Abstract

**Background:**

Biomedical researchers are now often faced with situations where it is necessary to test a large number of hypotheses simultaneously, eg, in comparative gene expression studies using high-throughput microarray technology. To properly control false positive errors the FDR (false discovery rate) approach has become widely used in multiple testing. The accurate estimation of FDR requires the proportion of true null hypotheses being accurately estimated. To date many methods for estimating this quantity have been proposed. Typically when a new method is introduced, some simulations are carried out to show the improved accuracy of the new method. However, the simulations are often very limited to covering only a few points in the parameter space.

**Results:**

Here I have carried out extensive *in silico* experiments to compare some commonly used methods for estimating the proportion of true null hypotheses. The coverage of these simulations is unprecedented thorough over the parameter space compared to typical simulation studies in the literature. Thus this work enables us to draw conclusions globally as to the performance of these different methods. It was found that a very simple method gives the most accurate estimation in a dominantly large area of the parameter space. Given its simplicity and its overall superior accuracy I recommend its use as the first choice for estimating the proportion of true null hypotheses in multiple testing.

## Introduction

In this genomic era, biomedical researchers are often faced with situations where a large number of hypotheses (a few thousands to tens of thousands) are being tested simultaneously. For example in a high-throughput microarray-based comparative gene expression study, a hypothesis concerning the differential expression status of each gene between two or more biological conditions is tested, and this is applied to all the genes being measured by the microarrays. The result of each statistical hypothesis testing is a p-value summarizing the level of statistical significance of the observed differential gene expression. With so many null hypotheses being tested simultaneously, the conventional single hypothesis testing criterion (p-value
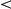
0.05) to reject a null hypothesis and declare significance in the result is no longer adequate, because too many false positives become inevitable. Take the microarray-based comparative gene-expression studies for example, suppose the two biological conditions being compared are the same, so no gene is differentially expressed. However, if 10,000 genes are being measured by the microarrays, on average 500 of these genes (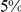) will turn out to have a p-value
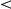
0.05, purely by chance. So an accurate estimation of the number of false positives is very important for researchers to correctly interpret the results and it is often necessary to control the number of false positives among the claimed significant results.

The classical method for false positive error control in multiple testing is the Bonferroni procedure, which controls the family wise error rate (FWER) at 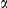 by setting threshold p-value as 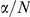, where 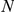 is the total number null hypotheses being simultaneously tested. However, the Bonferroni procedure is now widely recognised as being too conservative and leading to high rate of false negatives. The FDR (false discovery rate) approach has gained much popularity in recent years as it is more pertinent to the biomedical problems encountered in the high throughput omics era. In microarray gene expression studies, for example, researchers are naturally more interested in the proportion of false positives among the genes that are declared as significant. There are several closely related yet mathematically different measures in the literature, which were all intended to describe and quantify the proportion of falsely rejected null hypotheses among all rejected ones, namely, FDR (False Discovery Rate), pFDR (positive FDR), mFDR (marginal FDR), eFDR (empirical FDR), and cFDR (conditional FDR), the detailed definitions of which can be found, for example, in [1]–[3]. In this paper, I approach the multiple testing problem by trying to estimate the expected number of false positives 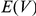 rather than the above error rates directly. Once 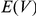 has been estimated, it is relatively straightforward to calculate mFDR and eFDR because these two error rates are less entangled than FDR or pFDR. Fernando *et al* showed that mFDR, or PFP (proportion of false positives) as they referred to it, have two desirable properties that FDR, pFDR, FWER do not possess; they also demonstrated that controlling these 3 error rates (FDR, pFDR, and FWER) does not necessarily lead to the control of the proportion of false positives among all positive results [4]. For these reasons above, I prefer to use PFP (or equivalently mFDR) as a measure describing and quantifying false positive errors in multiple testing.

A key factor in the accurate estimation of 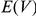 is the proportion of true null hypotheses 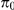 defined below, which is generally unknown *a priori*. Once this factor becomes known or is accurately estimated, it is then a straightforward step to estimate 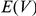, the expected number of false positives. For the control of various error rates such as FDR and pFDR, Black [5] pointed out that the main challenge in those procedures remains to be the accurate estimation of 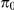, the proportion of true null hypotheses. So in this paper, our focus is on the accurate estimation of this key factor rather than on those different variants of FDR. Suppose in total 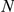 genes are being measured by microarrays and thus 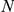 null hypotheses are being simultaneously tested. Suppose 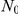 genes are non-differentially expressed, the proportion of true null hypotheses is hence 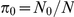. Given 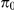 and a threshold p-value 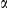, the expected number of false positives will be 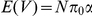. Thus by choosing an appropriate value of 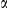, researchers can control the expected number of false positives as desired. Note that the formula 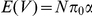 does not depend on the correlation structure among the 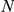 test statistics. In other words, these 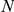 tests could be independent of each other, or correlated among themselves with any correlation structure. By focusing on the estimation of 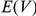 rather than the various error rates, we can reduce the multiple testing problem to the problem of estimating a single parameter 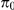. The performance of the methods used to estimate 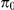 will generally be affected by the correlations among the test statistics, as will be shown in this paper.

Many methods for estimating 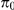 have been proposed in the literature, most of them require as input the p-values obtained from the 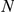 simultaneous statistical tests. In this study, I compare some commonly used methods and identify the ones that are significantly more accurate than others in different regions of the parameter space. I also make recommendations as to which method(s) should be used under some general and specific conditions.

## Methods

### Methods for estimating 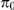


All the methods considered here for estimating the proportion of true null hypotheses require as input the p-values from the testing of 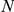 null hypotheses. Methods based on other strategies and requiring other type of data as input exist, for example, based on a re-sampling strategy [6], based on transformed test statistics [7], or based on estimating effect sizes [8]. However, these methods all require quite involved operations on the raw input data prior to or during the statistical testing step, and hence they are not as generally applicable to most situations as the p-value based methods. One of the advantages of the p-values based methods for estimating 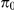 is their wide applicability, because the step of calculating p-values in statistical testing is well separated from the subsequent steps. Five different p-value based methods are considered in this study; the details of these methods can be found in the original publications that introduced them. Here I only give a brief description of each. Although the selection of these methods is far from exhaustive, they nevertheless represent some of the most commonly used ones in the literature. Their inclusion in this study was also partially due to the availability of software provided by the original authors implementing each method.

#### The PM03 method

Given 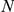 p-values obtained from testing 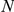 null hypotheses, the PM03 method [9] fits a beta-uniform mixture (BUM) model to the histogram of the observed p-values (empirical density distribution) with the following form of PDF (Probability Density Function)
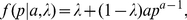
(1)where the first term 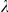 is for the uniform part of the mixture distribution model and the second term for the Beta part. The parameters 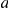 and 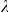 are first estimated by their maximum likelihood estimator (MLE) 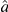 and 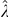, respectively, and then an upper bound of 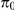 is estimated by 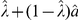.

#### The PC04 method

The PC04 method [10], instead of fitting a BUM model, applies LOESS to the p-value spacings to obtain an estimate of PDF, and takes the minimum value of the estimated PDF as an estimate of 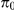.

#### The Ch04 method

The Ch04 method [11] uses an estimator for the CDF (Cumulative Distribution Function) of p-values in the form of a B-spline series with strategically designed knot sequence to achieve a desirable shape for the p-value cumulative distribution. The PDF is simply the first derivative of the CDF and the minimum of PDF is taken as an estimator of 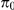.

#### The ST03 method

Exploiting the fact that p-values from true null hypotheses are uniformly distributed, the ST03 method [12] uses the following expression as an tunable estimate of 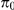


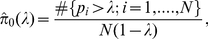
(2)with 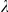 as the tuning parameter. It then fits a natural cubic spline with 3 degrees of freedom to the data of 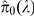 on some values of 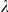, eg., 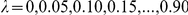, and finally the value of the fitted spline line at the end point 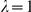 is taken as the estimate of 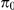.

#### The ZG04 method

In the ZG04 method [13], the 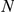 p-values are first sorted in ascending order so that 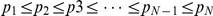. An empirical cumulative distribution of p-values is obtained by plotting 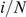 versus 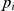. To estimate the proportion of true null hypotheses, the ZG04 method connects each point 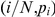 on the empirical cumulative plot to the end point 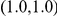 by a straight line, and calculates the slope of the line as
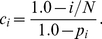
(3)Then the median value of 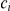 for a given range of 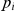, 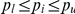, is taken as an estimate of 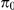,
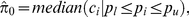
(4)with 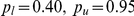 as the default values of the method parameters.

In this study, extensive *in silico* experiments are carried out to compare the performance of these 5 methods, with an unprecedented thorough coverage of the parameter space. The mean absolute error (MAE) is used as the main measure to compare the accuracies of these methods; the smaller the MAE, the more accurate the 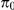 estimator. MAE is defined as
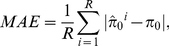
(5)where 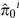 is the estimated value of 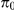 in the 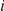
th *in silico* experiment, and 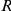 is the total number of such experiments for each point depicted in the parameter space. Another commonly used measure, RMSE (Root mean squared error), defined as
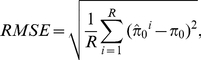
(6)could also be used to compare the accuracies of different methods. RMSE gives relatively higher weight to large errors, so it favors more accurate methods to stand out. MAE gives equal weight to all errors, and was felt to be more appropriate for this study. Note that both measures, MAE and RMSE, lead to the same conclusion in this study as to which estimator of 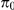 is overall the most accurate one.

### *In silico* experiment setup

Suppose we are carrying out a comparative gene expression study between two biological conditions, a control and a treatment. A gene's expression in the control condition is normally distributed as 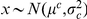, and in the treatment condition this gene's expression is distributed as 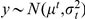, where the superscripts/subscripts 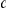 and 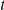 indicate the control and treatment condition, respectively. For simplicity let us assume that the variances of a gene's expression for the two conditions are the same, ie, 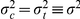. If a gene's mean expression levels in the two conditions are the same, ie, 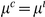, we say that this gene is non-differentially expressed, otherwise it is a differentially expressed gene. For a differentially expressed gene, we say it is up-regulated in the treatment condition if 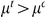, and down-regulated if 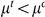.

**Figure 1:**
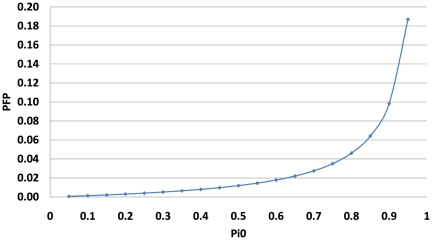
PFP as a function of 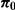 ranging from 0.05 to 0.95. For the *in silico* experiments with sample size 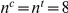, significance level 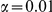 and typical effect size 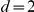, the power to detect such an effect is 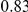 as can be seen from Table 1. The PFP will depend on the proportion of true null hypotheses 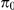 as shown in this figure.

In simulating a high-throughput comparative gene-expression study, 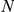 is the total number of genes being measured (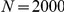 is used throughout these *in silico* experiments unless otherwise specified), and 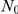 is the number of genes non-differentially expressed. For these 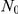 null genes (non-differentially expressed genes) I set 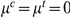 and 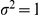 in generating their expression values. For each of the 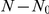 differentially expressed genes, 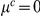 is set for the control condition and some non-zero value 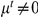 for the treatment condition. In this study, the 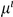 values are obtained by generating 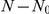 random numbers from a normal distribution 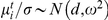 with 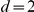 and 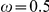 unless otherwise specified. So the typical effect of differential gene expression in this study is 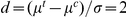. In order to achieve a reasonable power of statistical testing, a power and sample size calculation was carried out using a tool developed previously [14]. Although this tool was primarily designed for experiments where sample-pooling was involved, by setting the pooling parameters properly it can also be used as a general power and sample size calculation tool for two-sample t test with equal variance. For the power and sample size calculation in this study, 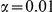 was used as a threshold for p-values, meaning that any observed differential gene-expression with a p-value less than 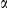 will be declared as significant. The results of this power and sample size calculation exercise are included in Table 1, which lists the statistical power achievable as the sample size increases. In most cases in the *in silico* experiments, 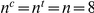 is chosen as the sample size. For a typical effect size of 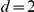 and a multiple testing situation where 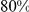 of genes are non-differentially expressed, this is the minimum number of arrays that would allow us to achieve a power above 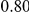 and a PFP below 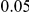, as can be seen from this Table. Increasing the sample size to 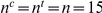 will increase the power to 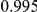, but the decrease in FPF is only 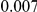. When the proportion of true null hypotheses 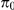 varies, the actual PFP varies accordingly. Fig. 1 shows PFP as a function of 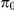, indicating that even when the proportion of true null hypotheses is 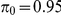, the PFP is still under 0.2. In other words, even when 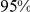 of the genes are non-differentially expressed, the *in silico* experimental setup would still allow us to achieve a reasonable PFP of below 0.2. So 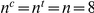 is chosen as the sample size for the majority of these *in silico* experiments; the guidelines being that the statistical power achievable in such experiments should not be too low or too high. Low powers are undesirable in real experiments and should be avoided at the experimental design stage; Too high the powers, on the other hand, can be too expensive and unrealistic in real microarray experiments. Here in this paper, the focus is on comparing the relative performance of several different methods under a typical experimental situation with a reasonable sample size and statistical power. In addition, the effects of varying sample sizes on the performance of these 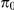-estimating methods are also investigated.

**Table 1:** The statistical power achievable in the *in silico* experimental setup.

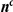	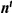	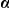	d	power	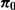	PFP
3	3	0.01	2	0.157	0.8	0.203
4	4	0.01	2	0.313	0.8	0.113
5	5	0.01	2	0.477	0.8	0.077
6	6	0.01	2	0.623	0.8	0.060
7	7	0.01	2	0.739	0.8	0.051
8	8	0.01	2	0.826	0.8	0.046
9	9	0.01	2	0.888	0.8	0.043
10	10	0.01	2	0.929	0.8	0.041
11	11	0.01	2	0.957	0.8	0.040
12	12	0.01	2	0.974	0.8	0.039
13	13	0.01	2	0.985	0.8	0.039
14	14	0.01	2	0.991	0.8	0.039
15	15	0.01	2	0.995	0.8	0.039

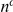
 and 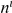 are the numbers of microarrays for the control and treatment conditions, respectively; 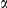 is the significance level of the statistical tests on genes' differential expression; 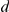 is the typical effect size for differential gene expression. PFP proportion of false positives.

### All genes are independent

First, consider the situation where all the 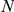 genes, whether differentially or non-differentially expressed between the two biological conditions, are independent of each other, meaning that a gene's expression is not influenced by or correlated to any other genes. This of course is not the closest representation of a real gene expression study, but might be an adequate theoretical model for the purpose of identifying individual differentially expressed genes. Importantly this is the theoretical basis upon which most of the 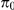-estimation methods were derived. So the first task is to compare these methods under this rather simplified assumption.

### Genes are correlated within groups

Next, it is interesting to see how these methods can be stretched to the regions where they were not initially designed for, how much deterioration would accumulate, and to compare the relative performance of these 5 methods when the above simplified assumption does not hold. Consider the situation where the genes are not all independent of each other, but they are correlated within some small groups. For simplicity in the simulations, a very simple correlation structure within a small group of genes is used. Note that in real experimental conditions, the correlation structures among genes can be much more complex and variable. In the simulation the 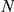 genes are divided into many small groups of 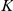; the gene groups are still independent of each other, but within each group the 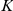 genes are correlated with the following 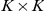 covariance matrix.
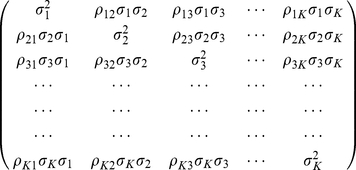
(7)In the covariance matrix (7), 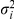 is the variance of gene 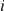
's expression; 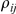 with 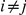 is the correlation coefficient between gene i and gene j within the group. By definition the correlation coefficient 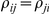. In this paper, the simplest covariance matrix with 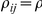 for all 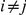 is used. In other words, all the pairwise correlation coefficients assume the same value 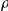. Thus by changing the value of 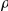 from 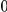 to 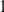, the correlation strength among the 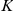 genes within a group can be tuned. The correlated expression values for the 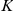 genes in a group can be generated using a multivariate normal distribution generator via a Cholesky decomposition of the above covariance matrix. Used in this work is the Java class *umontreal.iro.lecuyer.randvarmulti.MultinormalCholeskyGen* included in the SSJ Java library for stochastic simulation (http://www.iro.umontreal.ca/~simardr/ssj/indexe.html). Note that in dividing the total 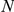 genes into groups of 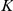, differentially expressed genes and non-differentially expressed genes are not put into the same group, as it is hard to imagine how such a correlation structure exists in real experiments. The reasoning is that if a gene's expression is affected by the treatment condition, any other genes correlated with it must also be affected by the treatment condition and hence they too should be differentially expressed. So genes are divided into many small groups, each consisting of 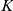 genes (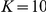 is used in all the *in silico* experiments unless otherwise specified) which are either all null or all differentially expressed. Genes from different groups are still independent of each other. The genes in the same group could be considered as being in the same pathways or involved in the same biological processes.

## Results

### All genes are independent

**Figure 2:**
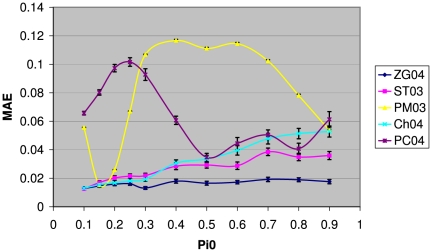
The mean absolute errors (MAE) of the 5 methods for different values of 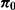. The error bar at each data point is the standard error of MAE based on 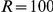 *in silico* experiments. Genes are assumed to be independent of each other. Overall the results here show that the most accurate method is ZG04 which has significantly lower MAE than the other methods for a wide range of values of 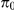. This figure also shows that ZG04, ST03 and Ch04 are much more stable than PM03 and PC04 whose MAEs fluctuate with changing 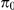.

The results of comparing the 5 methods under the independence assumption are shown in Fig. 2, where the mean absolute errors (MAE) for these methods are plotted as a function of 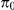. As can be seen from this figure, the accuracies of the PM03 method (yellow line) and PC04 method (purple line) are rather variable, with much wider ranges of MAE (changing from under 0.02 to above 0.1 for the PM03 method) as compared to the other 3 methods. The ST03, Ch04, and the ZG04 methods are more stable performers as the value of 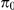 changes. There is a clear trend that both the ST03 and Ch04 methods become less accurate as 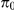 increases; however the magnitude of change in their MAE is relatively small compared to the PM03 and PC04 methods. This figure clearly shows that the ZG04 method is the most stable and accurate performer among these 5 methods, and is a clear winner with its MAE consistently lower than the other methods when 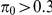. For small values of 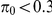, the separation of the ZG04 method from others become non-significant. The overall accuracy of the ZG04 method does not seem to be affected much as 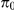 increases.

### Genes are correlated within groups

**Figure 3 pone-0018874-g003:**
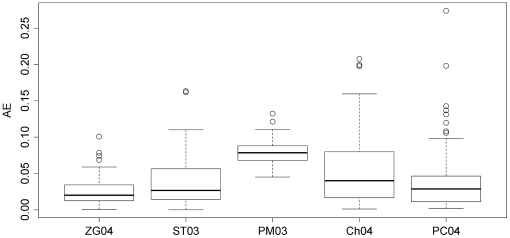
Box plot of the Absolute Errors (AE) of the 5 methods at 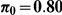 and 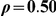. Based on the median AE, ZG04 is the most accurate among the 5, followed by ST03,PC04, Ch04, and PM03, in descending order of accuracy.

In addition to the simulations where all genes were assumed to be independent, extensive *in silico* experiments were also carried out with the block correlation structure described in the previous sections. The performance of these 5 methods was investigated with an unprecedented coverage over the parameter space 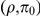. As an example, Fig. 3 shows the results for one point 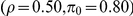 in the parameter space, based on 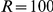 replicate *in silico* experiments. As can be seen from this figure, ZG04 is the most accurate method with the smallest median value of Absolute Errors (AE). ZG04 is followed by ST03,PC04, Ch04, and PM04 in ascending order of median AE. When the mean absolute error (MAE) was used as a measure of accuracy, ST03 and PC04 swapped places, resulting in the sequence ZG04, PC04, ST03, Ch04, and PM04 in ascending order of MAE. A two-sample paired t-test was carried out to compare the MAEs of the ST03 and PC04 methods. A p-vlaue = 0.99 was obtained, indicating that difference between these two methods was not statistically significant. All the other 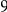 possible pair-wise comparisons among the 5 methods (ZG04 vs ST03, ZG04 vs PM03, …, PM03 vs Ch04, and Ch04 vs PC04, etc) using two-sample paired t-tests were also carried out; all those 9 comparisons yielded a p-value
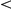
0.005, indicating that the differences in their accuracies were statistically significant (even under the very conservative Bonferroni multiple testing procedure). So the overall conclusions at this particular point 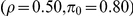 are: ZG04 is significantly more accurate than all the other 4 methods; ST03 and PC04 achieved statistically equivalent level of accuracy, and they both are significantly more accurate than the Ch04 and PM03 methods; and finally, the Ch04 method is significantly more accurate than the PM03 method at this point.

**Figure 4:**
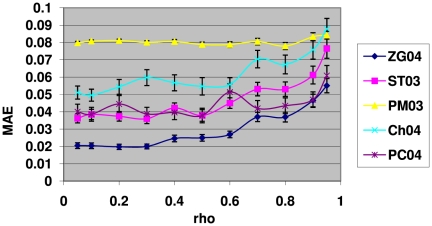
The mean absolute errors (MAE) of the 5 methods for 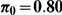 with different levels of inter-gene correlation. Overall the results here show that the most accurate method is ZG04 which is consistently and significantly more accurate than all the other methods for 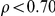. Beyond 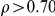, the difference between ZG04 and PC04 becomes statistically non-significant. The results here also clearly show that as the correlation strength increases, the ZG04 and ST03 methods become slightly but clearly less accurate. The PC04 and Ch04 methods seem to have similar trends but less clear-cut. The PM03 method is not affected much by the increasing level of inter-gene correlation, as its MAE values are almost flat with increasing 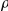.

Next it is interesting to investigate how these methods perform with varying strength of the inter-gene correlations 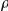 at a given value of 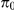. Fig. 4 shows the results of mean absolute errors for the 5 methods as a function of the correlation strength 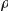 with the proportion of true null hypotheses fixed at 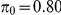. As can be seen from this figure, the ZG04 method, again, is the overall best performer, with its MAE value consistently lower (clearly so for 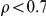) than all other 4 methods. There seems to be a trend for all 5 methods that when the inter-gene correlation strength increases, they all become less accurate to some extent. This is probably understandable as most of these methods were developed under the inter-gene independence assumption. Overall the deterioration in their accuracies as correlation kicks in and increases is not much, eg., 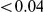 for the ZG04 and ST03 methods, which seem to be affected the most among these 5 methods.

### Global view in the parameter space 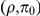


**Figure 5:**
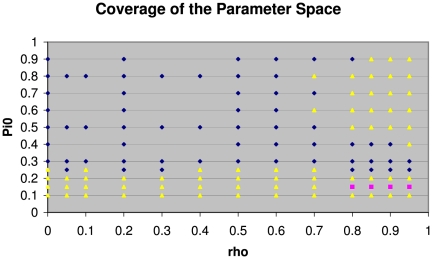
The 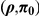 parameter space covered in this study by *in silico* experiments. For each data point marked on this graph, 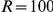 replicate *in silico* experiments were performed, and in each such experiment, 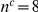 and 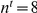 are the numbers of microarrays for the control and treatment conditions respectively. For each microarray, 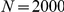 genes' expression values were generated. Blue diamonds indicate that the ZG04 method is significantly more accurate than all the other 4 methods. Yellow triangles: There is at least one other method achieving equivalent accuracy as the ZG04 method; Red squares: At least one method achieved a higher accuracy than the ZG04 method. The significance level was set at 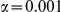, which controls the expected number of falsely significant claims at 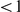. As there are about 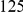 data points, each with four statistical tests, giving the total number of tests about 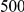.

To get a whole picture on the relative performance of these methods, systematic and extensive *in silico* experiments were carried out covering an unprecedented wide region of the parameter space. In total over 20 GB of data were generated counting all the simulated gene-expression data, statistical testing data, and the data on 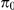 estimations and comparisons. The results of these systematic *in silico* experiments are summarised and shown in Fig. 5. Each marked data point in this figure represents 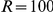 *in silico* replicate experiments with the parameters 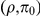 specified by the coordinates of that point. At each of the data points shown in this figure, gene-expression data were generated for 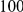 replicate *in silico* experiments, and then for each such *in silico* experiment, a two-sample T test with equal variance was applied to each gene, giving a p-value indicating the statistical significance of this gene between the control condition and the treatment condition. The vector of p-values for the 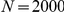 genes were then fed to these 5 different methods to estimate the value of 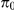. Because these were *in silico* experiments and the true value of 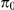 was known, so it was possible to assess the accuracies of these different methods by comparing their mean absolute errors as defined by Eq.(5). As there were 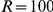 replicate *in silico* experiments at each 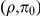 data point, statistical tests could be applied to four pair-wise comparisons between the ZG04 method and the other 4 methods. Given the results in Figures (2–4) that the ZG04 method was consistently more accurate than the other methods, it was important to investigate whether this holds true across the 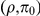 parameter space. Now that the emphasis was to compare the ZG04 method with others, only the following four pair-wise comparisons were performed: ZG04 vs ST03, ZG04 vs PM03, ZG04 vs Ch04, and ZG04 vs PC04. A two-sample paired T test was applied to each of the 4 pair-wise comparisons, and a threshold p-value 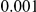 was used to declare statistical significance. There are 3 possible mutually exclusive outcomes of these comparisons: First, if ZG04 method is significantly more accurate than the other method in all these 4 pair-wise comparisons, ZG04 method is called as most accurate at this point of the parameter space; the data point will be shown as a blue spot (diamond) in Fig. 5. Second, if there is any one method which is statistically more accurate than the ZG04 method, that data point will be shown as a red spot (square) in Fig. 5. The third possibility is that the difference in accuracy between the ZG04 method and at least one other method is not statistically significant. In other words, at least one other method has achieved the same level of accuracy as the ZG04 method. In such a case, this data point will be shown as a yellow spot (triangle) in Fig. 5. The reason for setting the threshold p-value at 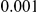 to declare statistical significance is the following. There are about 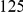 data points in the parameter space that were investigated. At each point, 4 statistical hypothesis tests are performed to compare these 5 methods, so the total number of tests carried out is about 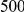. By setting the threshold p-value at 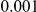, we expect to have less than 1 (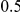 to be more precise) falsely declared significant results. In other words, by setting the threshold p-value as such, we are prepared to tolerate on average less than 1 falsely declared significance. This very stringent criterion ensures that when we say that one method is more accurate than another, we have the ultimate confidence that this is almost certainly true.

**Figure 6:**
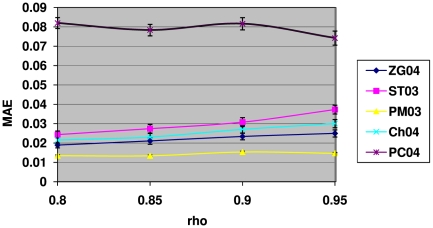
The PM03 method becomes more accurate in a small strip of the parameter space around 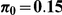 and 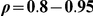. This figure shows that ZG04 is still the second most accurate method in this narrow strip.

As can be seen from Fig. 5, in a large bulk area of the parameter space 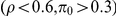 (the blue region), the ZG04 method is clearly the consistent winner. When the inter-gene correlation increases beyond 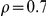 or when the proportion of true null hypotheses is relatively small 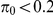, one or more other methods achieved statistically the same level of accuracy as the ZG04 method (in the yellow region). Also noticeable is that in a narrow strip of the parameter space (the red strip), one method became statistically more accurate than the ZG04 method, and it was the PM03 method. Shown in Fig. 6 are the the comparisons of the 5 methods in this narrow strip of the parameter space. Clearly the PM03 method achieved the lowest MAE, thus it was the most accurate method in this region. The performance of the PM03 method seems to be dependent on the value of 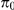, as also evident from Fig. 2 (yellow line), where it showed the biggest fluctuation with 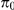 among the 5 methods. From that figure, one can see that near 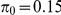, the MAE of the PM03 method quickly dropped to its minimum. The fact that the PM03 method is most accurate at around 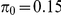 may suggest that this method should be used in this region. However, in actual applications, one would not know the true value of 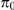, so a safe choice of the 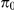-estimation method is to use one that is most accurate in the largest area of the parameter space, which is the ZG04 method as has been demonstrated in this study. If the estimated value of 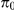 is in the neighborhood of 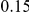, one can then use the PM03 method in order to achieve further a slightly more accurate estimation.

### Excursions into the sample size dimension

So far all the *in silico* experiments have been carried out with a fixed sample size, ie, 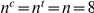. This choice of sample size was guided by the sample size and power calculations conducted in previous sections. One could argue that the global view depicted in Fig. 5 in the 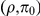 parameter space regarding the relative performance of these 5 methods was only based on this fixed sample size, hence its representative value is limited. So it would be interesting to investigate the effect of sample size on the performance of these 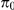-estimation methods. Given that the amount of simulations and time required to cover the current 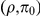 2-dimensional parameter space was already very extensive, to add another whole dimension (sample size 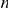) and to cover the 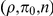 3-dimensinal parameter space would be too time-consuming and practically infeasible. So instead I chose some representative points in the 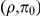 2-dimensional space, and made excursions into the 3rd dimension (the sample size dimension) to observe what effects these varying sample sizes have on the relative performance of the 5 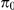-estimation methods.

**Figure 7:**
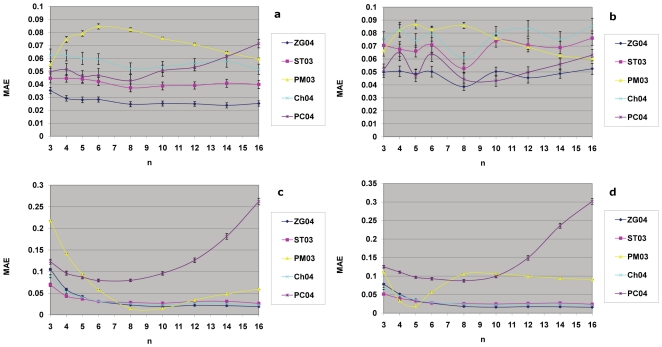
The effects of sample size variation on the accuracies of 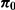-estimation methods. Shown here are the MAEs for the 5 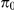-estimation methods as a function of sample size 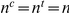. The results for four representative spots in the 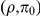 space show that as the sample size is varied, these spots remained predominantly their original behavior presented in Fig. 5. (a) Blue spot 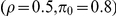 (b) Yellow spot 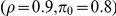 (c) Red spot 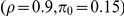 (d) Blue spot 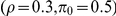.

The first point chosen was a blue spot in the 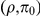 space 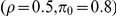. A series of simulations were carried out with different samples sizes while keeping other parameters fixed. As can be seen in Fig. 7a, the ZG04 method remained consistently the most accurate method with varying sample sizes. So the results here show that the spot at 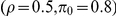 stays blue as the sample size varies; and it is especially so when the sample size increases.

The second point was a yellow spot 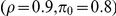, a spot where no clear winner was declared at 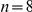, which meant that at least one other method achieved an equivalent level of accuracy as the ZG04 method, and the difference between them was not statistically significant. Fig. 7b shows the mean absolute errors (MAEs) of these 5 methods as a function of sample size 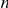. As can be seen from this figure, as the sample size varied the PC04 method stayed close to the ZG04 method; they competed and entangled; however their difference was not statistically significant due to the relatively large error bars. This point 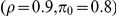 remained a yellow spot with varied sample sizes.

The third point was a red spot 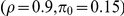 where at least one other method (the PM03 method) achieved higher accuracy than the ZG04 method when 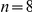. Fig. 7c shows the performance of the 5 methods under the influence of varying sample sizes. Here at this point, ST03, PM03 and ZG04 in turn became the most accurate method as the sample size increased from 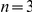 to 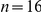. The color of this spot would change from red, to yellow, to red, to yellow, then to blue. This was because the point 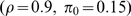 was close to the yellow-red boundary; it is understandable that its color was susceptible to mutation when the sample size varied. But overall this spot stayed dominantly as a red one.

The last point was a boundary blue spot 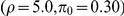, where ZG04 was the most accurate method for 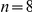. This spot was close to the blue-yellow boundary suggested that it might mutate its color as some other parameters varied. Fig. 7d shows MAEs of the 5 methods as a function of sample size n. Starting from 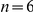 onwards ZG04 remained the most accurate method and this became statistically significant from 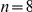 onwards, so this point stayed predominantly blue. It looks though that as the sample size is increased, the strong position of ZG04 method is reinforced; and it is weakened when the sample size becomes too small. However in real experiments, small sample size is not a desirable direction to go; particularly the statistical power achievable is too low (less than 0.5 when sample size is less than 6, as shown in Table 1), hence small sample size should be avoided in real experiments at the experimental design stage.

Overall, the view in the 2-dimensional 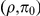 parameter space provided a reasonably accurate picture regarding the relative performance of these 5 methods, and it is of good representative value. Even when the sample sizes were varied, the four representative points investigated here demonstrated that the results shown in Fig. 5 largely persisted, especially when the sample size was increased, which is the desirable direction in real experiments.

## Discussion

In this study we have made the observation that the ZG04 method outperforms the other 4 over a large region of the 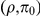 parameter space. To understand what makes this method perform better, let's compare the ZG04 method with the ST03 method. Both methods try to exploit the fact that p-values arising from true null hypotheses are uniformly distributed over 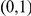. The ST03 method first fits a natural cubic spline based on about 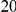 point estimates of Eq.(2) (The default setting for the ST03 method was using the sequence of 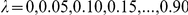), and then takes the value of the fitted spline at 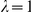 as the final 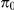 estimate. The ZG04 method, on the other hand, makes a point estimate of 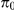 by Eq.(3) for every 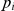 in the range 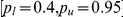 (The default setting of the ZG04 method). The number of 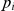
s within this default range of 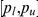 is usually in the order of 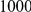, which means that the ZG04 method is based on much more point estimates than the ST03 method. This may help explain the potential of the ZG04 method to outperform the ST03 method. Secondly, the upper half of the p-value range, namely 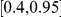 (the ZG04 default setting), may be more relevant and efficient than the whole range 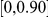 (the ST03 setting) in estimating 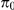, because the upper half of the p-value range tends to be populated less with p-values arising form non-null hypotheses. In other words, the distribution of p-values in the upper range reflects mostly those p-values arising from the true null hypotheses. In devising the ZG04 method, three possible measures could be used as the final estimate of 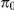, namely the minimum, the mean, and the median of the calculated slopes Eq.(3) for the given range 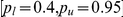. Given that the min tended to underestimate the true values of 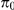 [14], the median was adopted because it was less sensitive to extreme values and outliers. The fact that the median is more robust than the minimum or the mean may also help to explain the consistent performance of the ZG04 method. It is a necessary practice to tune some parameters (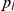 and 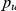 in the case of the ZG04 method) and to choose the most appropriate measure (the median of the slope 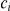
's) to achieve the best performance at the method-devising stage. But once this has been done, it is only fair to compare different methods at their respective default settings and it is probably the only feasible way to compare several different methods extensively. And this is what have been carried out in this study. It is very difficult to present a thorough analysis for each pair of methods explaining why one method is more accurate than the other. Nevertheless, the above analysis comparing ZG04 and ST03 hopefully can give some sense of why the ZG04 method outperforms the ST03 method. The PM03 method uses a beta-uniform mixture model to fit the histogram of p-values. The fact that PM03 performs best near 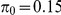 may suggest that this is an area of the parameter space where the empirical distribution of p-values is best described by the BUM model, which however becomes a less accurate model over other parameter regions.

It should be noted that this *in silico* study has been very extensive in terms of its coverage over the 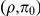 parameter space and its data volume. If it were a real experimental study, it would be equivalent to microarray experiments with about 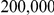 microarrays. To the best of my knowledge, no other similar simulation studies with this magnitude have been reported yet in the literature. Although very extensive, this study is still far from comprehensive because there are a couple of important aspects that need to be addressed, which are beyond the scope and capacity of a single study. Here in this paper I only touch on and briefly discuss them.

The first aspect concerns the number of different methods included under this study. Given the amount of simulations and subsequent analysis required in such an extensive study, my strategy in carrying out the investigation was to focus on a few most-commonly used methods, and get an overall picture regarding their relative performance. The number of methods included in this study may be relatively small, but the remit of this work is well-defined and focused. This forms a solid base for any future investigations to incorporate the findings established here. For example, any other methods or new methods only need to be compared with the best performing methods identified here, i.e., the PM03 method around the 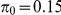 region, and/or the ZG04 method over a large region of the parameter space.

Another important aspect concerns the influence of the underlying gene-expression distributional assumption on the accuracies of these 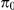-estimation methods. All the methods investigated here are p-value based methods, the advantage of which is their flexibility and wide applicability. They only require a vector of properly calculated p-values as input. The underlying distributional form of the gene-expression values and the type of statistical test used are shield away from these 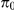-estimation methods themselves by the p-values. In this study I have used a single distributional assumption, i.e, the normal distribution, as the underlying distribution for the gene expression values. For this distribution, the two-sample t test is the proper statistical test to use. As the focus of this study is the estimation of 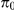 given a vector of properly calculated p-values, it is adequate to use the normal distribution as a most convenient instrument to generate the gene expression values and then to use its proper form of statistical test to calculate the p-values. It would be interesting to see how the underlying distributions affect the accuracies of these 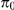-estimation methods, especially how robust these p-value based methods are when the actual distribution of gene expression departs from the assumed theoretical distribution. This would form the basis of a future investigation, which is beyond the scope of the current study.

In conclusion I have carried out extensive comparisons of some commonly used methods for estimating the proportion of true null hypotheses in multiple testing. The coverage of these *in silico* experiments over the 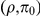 parameter space is unprecedented, thus enabling us to get a global picture on the relative performance of these methods. I identified ZG04 as the most accurate one among the 5 methods investigated over the largest area of the parameter space. Overall, based on the evidence gathered here in this study, I recommend the ZG04 method as the first choice for estimating 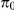. In a vast majority of times, the ZG04 method will give the most accurate results (the blue region) among the 5 methods, or at least achieving an equivalent accuracy as others (the yellow region). In a narrow strip of the parameter space around 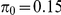, the PM03 method becomes more accurate than the ZG04 method, so I also recommend its subsequent use if the ZG04 method gives an estimation of 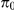 in the region 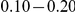. This hopefully will further increase the high accuracy already offered by the ZG04 method. Given the widely recognized paramount importance of the 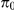 parameter in multiple testing, all the efforts towards more accurate estimation of 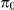 are greatly helpful to the subsequent calculations or controls of various error rates, such as PFP, FDR, pFDR, and eFDR.
